# Revisiting Konzo Risk Factors in Three Areas Differently Affected by Spastic Paraparesis in Eastern Democratic Republic of the Congo Discloses a Prominent Role of the Nutritional Status—A Comparative Cross-Sectional Study

**DOI:** 10.3390/nu13082628

**Published:** 2021-07-29

**Authors:** Marius Baguma, Espoir Bwenge Malembaka, Esto Bahizire, Germain Zabaday Mudumbi, Dieudonné Bahati Shamamba, Alain-Narcisse Matabaro, Jean-Michel Rigo, Alfred Kongnyu Njamnshi, Joelle Nsimire Chabwine

**Affiliations:** 1 Faculty of Medicine, Université Catholique de Bukavu (UCB), Bukavu 285, Democratic Republic of the Congo; bwenge.malembaka@ucbukavu.ac.cd (E.B.M.); esto.bahizire@gmail.com (E.B.); joelle.chabwine@unifr.ch (J.N.C.); 2 Department of Internal Medicine, Hôpital Provincial Général de Référence de Bukavu (HPGRB), Bukavu 285, Democratic Republic of the Congo; 3 Biomedical Research Institute (BIOMED), Faculty of Health and Life Sciences, UHasselt—Hasselt University, 3590 Diepenbeek, Belgium; jeanmichel.rigo@uhasselt.be; 4 Center for Tropical Diseases & Global Health (CTDGH), Université Catholique de Bukavu (UCB), Bukavu 285, Democratic Republic of the Congo; 5 Department of Epidemiology, Johns Hopkins Bloomberg School of Public Health, W6030, Baltimore, MD 21205, USA; 6 Center for Research in Natural Sciences of Lwiro, Bukavu 285, Democratic Republic of the Congo; 7 Department of Medical Microbiology, University of Nairobi, Nairobi 00100, Kenya; 8 Department of Pediatrics, Hôpital Provincial Général de Référence de Bukavu (HPGRB), Bukavu 285, Democratic Republic of the Congo; germainmudumbi@gmail.com; 9 Plant Pathology Laboratory, Faculty of Agronomy, Université Catholique de Bukavu (UCB), Bukavu 285, Democratic Republic of the Congo; bahati.shamamba@gmail.com; 10 Department of Medical Biology, Hôpital Provincial Général de Référence de Bukavu (HPGRB), Bukavu 285, Democratic Republic of the Congo; bionarcis.cd@gmail.com; 11 Department of Internal Medicine and Specialties/Neuroscience, Faculty of Medicine and Biomedical Sciences, The University of Yaoundé I, Yaoundé P.O. Box 25625, Cameroon; alfred.njamnshi@brainafrica.org; 12 Brain Research Africa Initiative (BRAIN), Yaoundé P.O. Box 25625, Cameroon; 13 Brain Research Africa Initiative (BRAIN), Thônex, 1226 Geneva, Switzerland; 14 Neurology Unit, Department of Neuroscience and Movement Science, Faculty of Science and Medicine, University of Fribourg, 1700 Fribourg, Switzerland

**Keywords:** cassava, cyanide poisoning, konzo, malnutrition, South-Kivu

## Abstract

This comparative cross-sectional study aimed to better understand the respective contributions of protein malnutrition and cassava-derived cyanide poisoning in the development of konzo. We compared data on nutritional status and cyanide exposure of school-age adolescent konzo-diseased patients to those of non-konzo subjects of similar age from three areas in the Eastern Democratic Republic of the Congo. Our results show that konzo patients had a high prevalence of both wasting (54.5%) and stunting (72.7%), as well as of cyanide poisoning (81.8%). Controls from Burhinyi and those from Idjwi showed a similar profile with a low prevalence of wasting (3.3% and 6.5%, respectively) and intermediate prevalence of stunting (26.7% and 23.9%, respectively). They both had a high prevalence of cyanide poisoning (50.0% and 63.0%, respectively), similar to konzo-patients. On the other hand, controls from Bukavu showed the lowest prevalence of both risk factors, namely chronic malnutrition (12.1%) and cyanide poisoning (27.6%). In conclusion, cassava-derived cyanide poisoning does not necessarily coexist with konzo outbreaks. The only factor differentiating konzo patients from healthy individuals exposed to cyanide poisoning appeared to be their worse nutritional status. This further suggests that, besides the known role of cyanide poisoning in the pathogenesis of konzo, malnutrition may be a key factor for the disease occurrence.

## 1. Introduction

Konzo is a tropical toxico-nutritional spastic paraparesis (SPP) associated with sulfur amino acid (SAA) deficiency and monotonous consumption of toxic cassava products [1]. The diagnosis of konzo is currently made strictly on a clinical basis, using the World Health Organization (WHO) criteria [1]: (1) a visible symmetric spastic abnormality of gait while walking or running; (2) a history of onset of less than 1 week, followed by a non-progressive course in a formerly healthy person; and (3) bilaterally exaggerated knee or ankle jerks, without signs of disease of the spine. Depending on the severity of the disease, patients suffer from para- or tetraparesis. They are able to walk (spastic gait) without aid (mild forms), with one or two sticks (moderate forms), or either use a wheelchair or are bedridden (severe cases) [2,3].

Although only a low number of individuals suffer from konzo worldwide, this irreversibly handicapping disease is restricted to a few areas in Africa [3,4], where it becomes a prominent public health problem [5], with usual prevalence ranging from 1 to 5%, but rising at times up to 20%, as, for instance, in the Western Democratic Republic of the Congo (DRC) [6,7]. Konzo cases mostly occur as epidemic outbreaks in communities relying on cassava (*Manihot esculenta* Crantz) as the staple food and facing harsh conditions, such as drought, famine or war [8,9]. These conditions lead to a high prevalence of protein malnutrition and to dietary cyanide poising from the consumption of toxic, insufficiently processed, bitter cassava roots [4].

In the late 1990s, cases of SPP fulfilling the WHO clinical criteria for konzo appeared in Burhinyi, a rural area located in the province of South-Kivu in Eastern DRC [9]. Cassava-derived cyanide poisoning and poor nutritional status, well-known as konzo risk factors [3,4], were documented in affected patients, further confirming the etiology of this outbreak of SPP. Shortcuts in processing of (bitter) cassava products prior to consumption and food deprivation, most probably related to war events, appeared to be the main triggering factors [9]. Although no subsequent konzo outbreak has been reported in the region during the last decade, people in the area are still exposed to cyanide poisoning [10], and one to four sporadic cases are still recorded yearly. Furthermore, it is not clear why konzo cases are observed exclusively in Burhinyi and its immediate neighborhood, as (1) war events in Eastern DRC involved a region much larger than Burhinyi and (2) cassava remains the main caloric source in the whole province of South-Kivu (where bitter cassava is widely consumed). In addition, the first survey [9] lacked a comparison between data from SPP-affected patients and non-affected subjects, which could have given more insight into epidemic determinants.

From the abovementioned statements, we hypothesized that (1) konzo-affected patients would have worse nutritional status than non-affected subjects from Burhinyi, but they would similarly be exposed to cyanide poisoning from cassava, because they are submitted to the same diet; and (2) people from konzo-free areas with available alternative caloric and protein sources would have a better nutritional status and potentially undergo less cyanide intoxication from cassava, as no shortcuts would be needed in the processing of cassava. Thus, through careful assessment of supposedly different contextual levels of malnutrition and of cassava-derived cyanide exposure, this study aimed at a better understanding of the respective contributions of these risk factors in the occurrence or absence of konzo.

## 2. Materials and Methods

### 2.1. Study Areas

This study was conducted in the province of South-Kivu, in the eastern part of the DRC, characterized by a mountainous landscape, a dense hydrography and a humid tropical climate tempered by altitude, with an alternation of two seasons: a short dry season (June to August) and long rainy season (September to May). The whole region went through drought in the last two decades, supposedly due in part to deforestation [11,12], even if one could also question direct effects of climate change. In addition, since the late 1990s, the province, and particularly its rural areas, has been torn by war and violence, including mass killings and systematic rapes [13,14].

Subsistence agriculture and petty trading are the main economic activities in most rural areas of the province, while in cities, the economic activities include secondary and tertiary activities. In most areas, the diet is composed of a main dish (based on usual crops and tubers) and a side dish (made of meat, poultry, fish or vegetables, such as beans, peas, pounded cassava leaves, squash leaves, amaranth, etc.). The most popular (traditional) main dish is called “Ugali” or “fufu”, a kind of hard porridge obtained by kneading cassava flour in boiling water. Thus, cassava (flour) constitutes, as in other tropical areas, the most important source of calories, but it is deficient in proteins (especially in SAA) [15]. To compensate for cassava deficiency in proteins, some populations (mostly in cities such as Bukavu, where food variety is guaranteed) mix cassava with cereals to make flour for “Ugali” or use other main dishes such as rice, potatoes, or sweet potatoes. However, previous studies have documented a limited intake of animal proteins in rural areas over the whole DRC, and particularly in the province of South-Kivu [16,17].

In order to appropriately test and operationalize our hypotheses, we selected 3 different areas in the province: Bagira, Burhinyi and Idjwi. Bagira, being a health zone located in Bukavu, the capital-city of the province, will hereafter, be referred to as “Bukavu”. Bukavu is a small growing city of about 1 million inhabitants and is characterized by secondary and tertiary economic activities; the diet is based on food provided by neighboring villages. Thus, the earning power is assumed to be higher than in rural areas, and the nutritional status is better. Furthermore, because rural populations preserve cassava products of the highest quality (i.e., with the most appropriate processing) for trade, cyanide poisoning is supposedly minimal in Bukavu (where cassava is not cultivated). Burhinyi is a mountainous rural area located 100 km southwest of Bukavu, with subsistence agriculture and petty trading as its main economic activities, where konzo cases were first reported in Eastern DRC [9]. Idjwi is the largest island of the Kivu Lake, located 49 km to the northeast of Bukavu. The lifestyle is predominantly rural, but contrary to Burhinyi, the population in Idjwi does not rely solely on the standard diet described above. In fact, the Kivu Lake provides fish, which not only enriches the diet, but also improves the local economic power (via trade with neighboring cities). Furthermore, the soil is fertile, making various tubers and vegetables more available. Thus, the nutritional status in Idjwi is assumed to be higher than in other rural areas. Two additional specificities make Idjwi even more interesting for this study: contrary to Burhinyi, Idjwi island has been relatively spared from dramatic effects of recent wars in the region (probably in part due to geographical isolation), while, in contrast, cyanide toxicity from cassava was documented since the 1970s [18], without any case of konzo being reported so far.

Thus, by specifically selecting these three areas, we expected that Burhinyi participants (the only area where konzo was reported in the province) would demonstrate the worst nutritional status, contrary to Idjwi and Bukavu, whereas individuals from Burhinyi and Idjwi would undergo higher exposure to cassava-derived cyanide exposure in comparison to Bukavu. These different patterns of nutritional status and cyanide exposure could provide more insights into the respective contributions of these two factors on the development of konzo.

### 2.2. Study Design and Ethics

This comparative cross-sectional study was conducted essentially between November 2012 and June 2014, recording data on the nutritional status and cassava-related cyanide exposure in non-konzo children and adolescents of school-age, from the three areas of interest (i.e., Bukavu, Burhinyi and Idjwi). Additionally, similar data were extracted from previous reports [9] on konzo-diseased patients of similar age (exclusively from Burhinyi) for comparison purpose. Complementary information on cassava varieties and processing was obtained from a few adults selected purposively in the study area.

The study was approved by the ethical committee of the Université Catholique de Bukavu (ethical approval code: UCB/CIES/NC/015B/2014) and conducted according to the 1964 Declaration of Helsinki and its later amendments, and following national and international ethical standards. Prior to enrolment in the study, all adult participants gave their informed oral consent, while for those under the age of 18, the assent was obtained from parents.

### 2.3. Participants

Data of konzo patients were previously collected in two old surveys performed respectively in 2003 and 2005 (reporting first konzo in Burhinyi) [9]. It was not possible to include new patients because no new outbreak of konzo occurred since the first report, but only a few sporadic cases every year. Out of 41 konzo patients finally selected in the abovementioned investigations, only non-adult patients at the time of their disease (i.e., ≤18 years old, corresponding approximately to the school-age) were included in this study.

Controls were recruited in 2014 from the three areas described above (for details, see the Section 2.1, “Study Area”, in the Materials and Methods section). Exclusion criteria for controls consisted of smoking and presence of motor lower limb impairment. Due to contextual difficulties (such as access to participants, lack of funding, etc.) that did not allow us to match controls to the exact patients’ ages and sex with a predetermined proportion, we opted to target an age range close to that of patients in three high schools (10–18 years old, first to sixth year) located each respectively in one of the three areas of interest, using a pseudo-randomization procedure to minimize at best potential selection bias. Due to the same difficulties that strongly constrained the way the study was conducted, participants were conveniently included in order to obtain as larger groups as possible. By the means of alphabetically ordered lists obtained from school authorities, we selected each time students in one first year (names next to even numbers) and one fourth year (names next to odd numbers) class of one school in the area (school selection was determined by easiness of contact and permission from the school responsible to perform the study). The final sample size reached in each study area was as follows: 30 participants from Burhinyi, 46 from Idjwi and 58 from Bukavu. One subject from Burhinyi was excluded because he had a paraparesis of undetermined origin.

### 2.4. Data Collection

#### 2.4.1. General Data and Anthropometric Measures

All control subjects were interviewed on their medical history concerning malnutrition and spastic paraparesis (including other potential cyanide sources, such as smoking). In addition to a general physical examination, they were submitted to anthropometric measurements, using accessible standard tools. The weight was measured (in kg) to the nearest 0.1 kg accuracy, using an electronic balance scale (BS-branded 2005, max 150 kg, d = 0.1 kg). The height was measured (in cm) to the nearest round number, from the subject’s head (vertex) to the heel in an upright standing position, with five points of his body touching the wall, using a portable folding ruler (2 M branded, max 200 cm, d = 0.1 cm). For konzo patients, we used previously recorded anthropometric measures, which were obtained by following a similar procedure [9].

#### 2.4.2. Cassava-Derived Cyanide Exposure

In order to confirm cyanide exposure from cassava dietary products in Idjwi, we first collected information about consumption of bitter cassava in 13 adults (≥19 years old) conveniently selected in the neighborhood of the schools of control participants recruitment. They were interviewed on (1) the cassava varieties usually consumed in the local area, (2) their names in the local language, (3) the taste of consumed cassava (sweet or bitter) and (4) the common cassava processing methods. Second, samples of fresh cassava tubers were directly collected from local farms close to the participants’ houses in Idjwi. Collected cassava roots were stored in dry and cool conditions for a maximum of 48 h before their cyanide content was measured. Third, some tubers were experimentally processed by following the common procedure reported by interviewed people and cyanide levels measured on a daily basis for the whole duration of the processing. Data on cassava toxicity in Burhinyi were collected during a previous survey [9], following comparable procedures as for the present study (especially regarding cassava varieties, taste and root processing). We collected a few samples of cassava flour from different retailers in one Bukavu market, because cassava products consumed in Bukavu come from rural areas and are sold in markets mainly as flour.

In complement to the abovementioned data on cyanide content of cassava products, urinary thiocyanate (SCN) was measured as an in vivo marker of cyanide poisoning. For this purpose, all control participants gave urine samples collected in duplicate sterile 50 mL–tubes for each participant on the same day of the clinical examination. Fresh collected urine samples were immediately labeled with an anonymized code, put on ice in closed boxes for 48–72 h and, thereafter, deep-frozen until they were processed for urinary SCN measurements.

### 2.5. Data Processing and Analysis

#### 2.5.1. Data Processing

Nutritional status assessment was based on body mass index (BMI)-for-age and height-for-age indices. Wasting (indicating acute malnutrition) was defined by BMI-for-age index below −2 Z-score, and stunting (reflecting chronic malnutrition) was defined by height-for-age index below −2 Z-score of the reference value, using the WHO references for subject aged 5–19 [19,20]. Anthropometric indices were calculated with the WHO AnthroPlus software Version 1.0.4. (WHO, Geneva, Switzerland; Available online: http://www.who.int/growthref/tools/en/; accessed on 15 January 2019).

Cyanide content of fresh roots was measured by using semi-quantitative colorimetric methods with the “kit A”, according to Dr. Bradbury’s protocol (School of Botany and Zoology, Australian National University) [21]. The standard processing method described by interviewed people from Idjwi lasted 10 days in average (up to 14 days in case of rainy and humid weather). It consisted of 4 days of sun-drying, followed by 3 days of heap fermentation and finally 3 days of a second sun-drying session. This procedure was mimicked in the plant pathology laboratory of the Faculty of Agronomy of the Université Catholique de Bukavu (UCB), at the most identical, using similar material to spread and store cassava species under process. The experimental cassava processing was performed on two cassava varieties (respectively one bitter and one sweet variety), using 1 or 2 samples for each, and the cyanide concentration of each sample was measured every day, until it reached 0 ppm.

Urinary SCN concentrations were measured by using the “kit D1”, following Dr. Bradbury’s protocol (School of Botany and Zoology, Australian National University) [22], and the threshold for reference normal value was 100 μmol/L for individual with normal kidney function (corresponding to the cutoff value of 6 mg/g creatinine for non-smokers [23]). In this analysis, although we did not specifically check for kidney function, we did not have any clinical ground to suspect kidney failure in all recruited individuals. Therefore, we used the abovementioned reference value for measured urinary SCN in non-smokers.

#### 2.5.2. Statistical Analysis

Data are presented as frequencies and percentages for categorical variables, and as median with interquartile range (IQR) for continuous variables. Categorical data were compared by using Pearson’s Chi-squared test (or Fisher’s exact test when at least one expected cell size was <5), whereas quantitative data were compared by using the Mann–Whitney test or, when comparing all three control groups at a time, the Kruskal–Wallis test, corrected by Bonferroni’s multiple comparisons procedure. Statistical significance was defined by *p* < 0.05, and analyses were performed by using the Epi Info software, Version 7.2.4.0 (CDC, Atlanta, GA, USA, 2020) and Stata version 16.0 (StataCorp LLC, College Station, TX, USA, 2019).

## 3. Results

### 3.1. General Characteristics

In all, 145 subjects (44,8% females) aged 14 years (IQR: 13–15 years) years participated in this study (11 konzo patients, 30 control subjects from Burhinyi, 46 from Idjwi and 58 from Bukavu). Age was similar between participants from different areas (14 years (IQR: 12–16 years) for konzo patients, 13.5 years (IQR: 13–15 years) for controls from Burhinyi, 14.5 years (IQR: 13–16 years) for controls from Idjwi and 14 years (IQR: 14–15 years) for controls from Bukavu), while sex distribution differed: females predominated (63.6%) in konzo patients (exclusively originating from Burhinyi), while a majority of controls from Burhinyi (63.3%) and Bukavu (58.6%) were males. Females (50.0%) and males (50.0%) were equally prevalent in controls from Idjwi.

### 3.2. Nutritional Indices

**Figure 1:**
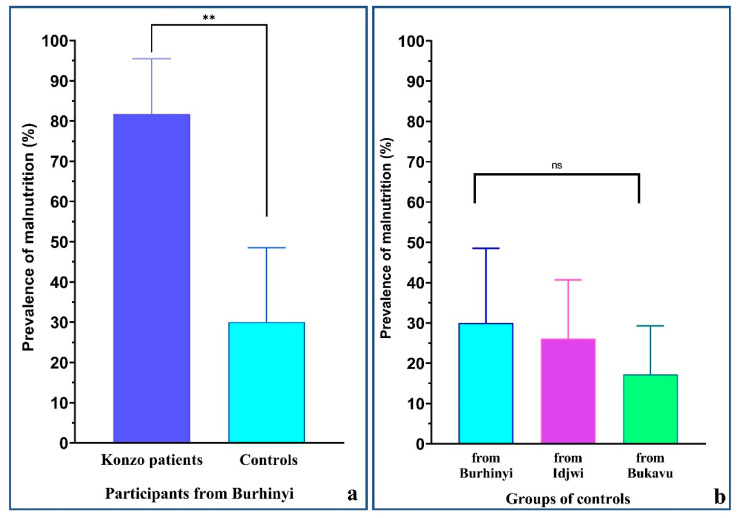
Prevalence of malnutrition in the studied population. This figure shows a significantly higher prevalence of malnutrition in konzo patients living in Burhinyi as compared to healthy controls from the same areas (*p* = 0.0046, Fisher’s exact test) (**a**). Furthermore, controls from Burhinyi tended to have a higher prevalence of malnutrition than those living in Idjwi or in Bukavu (*p* = 0.3407, Pearson’s chi-squared test) (**b**); ns, the difference is not significant. ** Statistically significant difference with *p* < 0.01.

Overall, konzo patients had both median BMI for age and height for age below −2 Z-score (respectively −2.2 (−2.6 to −1.6) and −3.2 (−3.7 to −1.8)), suggesting that they suffered from both acute and chronic malnutrition. In controls from the same area (Burhinyi), the height for age approached this threshold (−1.7 (−2.0 to −0.9)), while BMI for age was in normal reference range (−0.2 (−1.2 to 0.2)). Thus, there was a slight trend to chronic malnutrition, but no acute malnutrition. When comparing konzo patients with controls from Burhinyi, both acute and chronic malnutrition were significantly more prevalent in konzo patients than in controls (*p* = 0.0006 for wasting, and *p* = 0.0119 for stunting). Since the sample size was too small when discriminating between acute and chronic malnutrition, and for more reliable interpretation, acute and chronic malnutrition data were merged. However, still, the overall prevalence of malnutrition remained significantly higher in konzo patients than in controls from Burhinyi (81.8% vs. 30.0%, *p* = 0.0046) (Table 1 and Figure 1a).

Nutritional indices of controls from Idjwi were well above −2 Z-score (respectively 0.1 (−0.5 to 0.8) for BMI for age and −1.4 (−2.0 to −0.6) for height for age) and acute malnutrition had a low prevalence (<10%) in all control groups. Therefore, it was more convenient to compare the overall malnutrition prevalence. With respect to the latter, controls from Burhinyi tended to have a relatively higher prevalence (30.0%), as compared to those coming from Idjwi (26.1%) and from Bukavu (17.2%), but this difference was not statistically significant (Table 2 and Figure 1b).

In summary, konzo patients showed the worst nutritional status compared to controls from the same area and had a higher proportion of acute malnutrition. While the prevalence of acute malnutrition was low in all control groups, chronic malnutrition tended to be more present in those from Burhinyi; however, no statistical difference could be reached.

### 3.3. Cyanide Exposure from Cassava Products

#### 3.3.1. Cyanide Content of Cassava Roots and Flour

Three cassava varieties appeared to be predominantly consumed in Idjwi, two of which were bitter (namely Nambiyo and Mushikuzi in the local language). Fresh cassava root samples had median total cyanide levels of 200 ppm for bitter varieties and 30 ppm for the sweet variety. We could not analyze processed cassava roots from participants’ household stock, as only four of them brought samples, and we could not confirm whether those roots were for domestic consumption or for commercial purposes. The cyanide content of flour collected from the Bukavu market was within the safety range (median: 5 ppm, interquartile range: 0–5 ppm).

**Figure 2:**
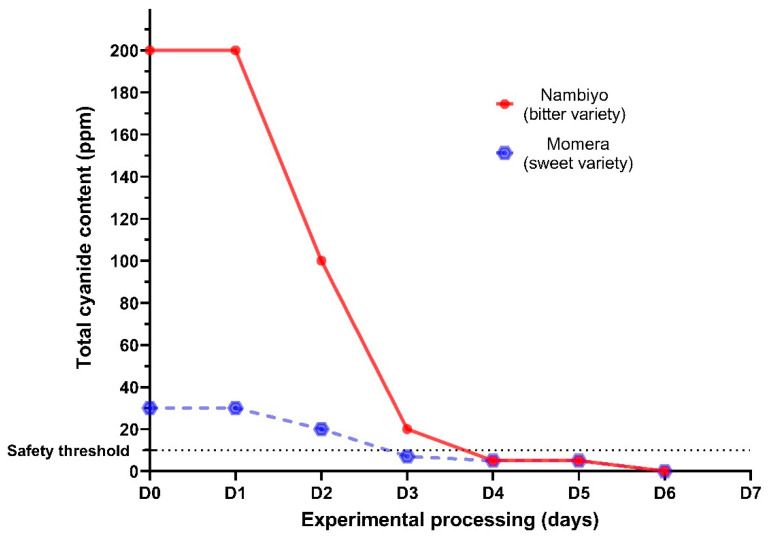
Results of the experimental processing of fresh cassava roots. From initial values of 200 ppm in the bitter cassava roots and 30 ppm in sweet ones, the total cyanide content in the fresh cassava roots progressively dropped below the safety threshold of 10 ppm on the fourth and the third days of processing, respectively. In both varieties, the cyanide content reached undetectable levels on the sixth days.

Fresh roots (1 or 2 samples) from one bitter (Mushikuzi) and one sweet (Momera) varieties were experimentally processed according to the standard procedure obtained from interviews, which lasted 10–14 days (see methods above). As shown in Figure 2, the initial cyanide content reached 200 ppm in bitter roots and 30 ppm in sweet ones; it then progressively decreased up to the safety threshold, reached on the fourth and the third processing days, respectively. Cyanide was no more detected on the sixth day, which halted the experiment for both cassava varieties.

#### 3.3.2. Urinary Thiocyanate Concentration

Overall, the median urinary SCN was almost double the reference value of 100 µmol/L in konzo patients (172.0 (103.2 to 344.0)), just exceeded it in controls from Idjwi (103.2 (34.4 to 172.0) µmol/L) and was close to it in controls from Burhinyi (86.0 (34.4 to 172.0) µmol/L). Controls from Bukavu had values well below the threshold (34.4 (17.2 to 103.2) µmom/L). Konzo patients had significantly higher median urinary SCN (*p* < 0.0001). Controls from Burhinyi and those from Idjwi had similar urinary thiocyanate levels (*p* = 0.2575), both significantly higher than controls from Bukavu (Burhinyi vs. Bukavu: *p* = 0.0026; Idjwi vs. Bukavu: *p* < 0.0001).

**Figure 3:**
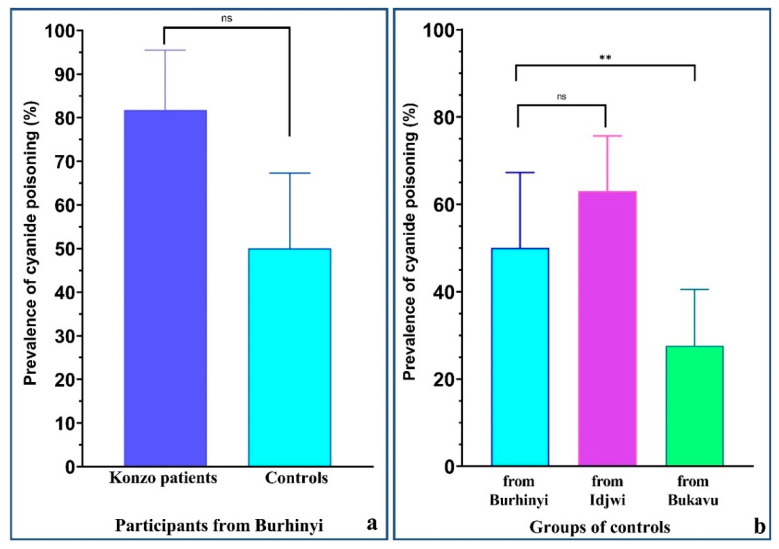
Prevalence of cyanide poisoning in the studied population. (**a**) 81.8% of konzo patients had urinary SCN above the reference value of 100 µmol/L, as compared to 50% of healthy controls from Burhinyi, but this difference was not statistically significant (*p* = 0.0855, Fisher’s exact test). (**b**) Controls from Burhinyi and Idjwi had similarly high prevalence of cyanide poisoning (50% and 63%, respectively). The prevalence of cyanide poisoning in these two groups was significantly higher than the one found in controls from Bukavu (27.6%), *p* = 0.0012 (Pearson’s chi-squared test). ns: the difference is not significant. ** Statistically significant difference with *p* < 0.01.

To better estimate how cyanide exposure was reflected by urinary SCN, it was more illustrative to evaluate the exact proportions of people with high urinary SCN (i.e., prevalence of urinary SCN above 100 µmol/L) in all groups and compare them. Konzo patients tended to have higher prevalence of excessive urinary SCN levels (81.8%) than in healthy controls from Burhinyi (50.0%), but the difference did not reach significance (*p* = 0.0855) (Figure 3a). Fewer controls from Bukavu had high urinary SCN (27.6%) compared to those from Idjwi (63.0%) and from Burhinyi (50.0%) (*p* = 0.0012), while there was no difference between controls from Burhinyi and from Idjwi (*p* = 0.2603) (Figure 3b).

In summary, konzo patients had a high prevalence of both acute and chronic malnutrition, as well as a high prevalence of cyanide poisoning. Controls from Burhinyi and those from Idjwi showed a relatively similar profile with low prevalence of acute malnutrition and intermediate prevalence of chronic malnutrition. They both had a high prevalence of cyanide poisoning, similar to konzo patients. On the other hand, controls from Bukavu showed the lowest prevalence of both risk factors, namely malnutrition and cyanide poisoning (Table 3).

## 4. Discussion

Almost a century since the first description of konzo, the causal agent(s) and pathogenic mechanism(s) of this disease remain unclear [4], while chronic cyanide intoxication and protein malnutrition are consistently found in konzo affected patients, constituting the main risk factors of konzo [3]. In addition, it is not known if both risk factors have the same contribution to the occurrence of the disease. To evaluate this question, we assessed the level of cyanide poisoning and the nutritional status of school-age adolescents from Burhinyi affected by konzo, as well as healthy subjects of similar age from three different areas of South-Kivu (Burhinyi, Idjwi and Bukavu). We hypothesized that konzo patients and controls from Burhinyi would have the worst nutritional status and highest cassava-derived cyanide exposure, while individuals from Idjwi would undergo similar cyanide exposure, but have a better nutritional level. Participants from Bukavu were assumed to have the best nutritional status, and no cyanide poisoning.

The most remarkable result of this study is the still ongoing cyanide exposure presumably originating from high cyanide-containing cassava products in Idjwi, an area where no case of konzo has been so far reported. Furthermore, the prevalence of high urinary SCN levels did not differ between participants from Burhinyi and those from Idjwi, confirming our initial hypothesis, but in contradiction with the existing literature, which suggests a difference of cyanide exposure between konzo-affected and non-affected areas [24,25,26]. Since the first outbreak of konzo in South-Kivu in the late 1990s [9] and the previously known cassava-derived cyanide poisoning in Idjwi supposedly contributing to endemic goiter [18], this is the first study comparing the toxicological profile and nutritional status of people living in these two areas.

The abovementioned finding questions the sole contribution of cassava-related cyanide poisoning in the development of konzo. In fact, the literature remains controversial about the difference of cyanide exposure between konzo-affected and non-affected individuals in areas of konzo epidemics. Our data, by showing no difference between people affected by konzo and non-diseased individuals, suggest that cyanide exposure is a necessary, but not a solely sufficient condition for the occurrence of konzo. Surprisingly, when comparing the overall nutritional status of konzo patients and controls from Burhinyi and from Idjwi, konzo patients showed significantly poorer nutritional status than both control groups, with no difference observed between the latter. Thus, the only factor differentiating konzo patients from non-konzo individuals exposed to cyanide poisoning appeared to be the worse nutritional status [27], further suggesting that, despite the known role of cyanide poisoning in the pathological mechanisms of konzo, malnutrition is a necessary factor for appearance of the disease. The differential contribution of konzo risk factors, as illustrated in Table 3, constitutes an innovative concept that brings new insights into understanding the disease determinants. Protein malnutrition (especially SAA deficiency) occurring in populations under severe food deprivation has been incriminated as a major nutritional factor involved in konzo [25,28]. Whether it occurs in an increasingly progressive but chronic way or more abruptly remains an open question. The idea of an abrupt/acute factor of undetermined nature triggering konzo outbreaks [29] favors the second alternative. The fact that no new konzo outbreak was observed in the province between the first report [9] and the present survey almost 10 years later, while there is no obvious indication that cyanide exposure and nutritional status dramatically deteriorated or improved in the meantime, suggests that a transient event that massively affected the population in Burhinyi led to the first outbreak. If we consider that, in our study, acute malnutrition was solely seen in the konzo group, and that prevalence of stunting was significantly higher in konzo-diseased patients than in controls [27], an acute worsening of the nutritional status (with presumable SAA deficiency) could have been one determining factor triggering konzo. Thus, overall, the contextual information and some indications from our results support the hypothesis of an acute factor triggering konzo epidemic that could be nutritional.

The prevalence of stunting was similar between controls from Burhinyi and from Idjwi. Hence, confirmation of the hypothesis discussed above would imply that any further deterioration of the nutritional status of Idjwi population would be at risk of triggering konzo. From a more general perspective, knowing the high prevalence of stunting in several African countries, and in particular those undergoing konzo outbreaks, this assumption would mean that a wider population is at risk of developing konzo. Therefore, careful monitoring and preventive strategies targeting specifically nutritional improvement in addition to promotion of safe cassava consumption should be advocated for in areas witnessing existence of konzo risk factors. In contrast, controls from Bukavu, not being exposed to cyanide toxicity and having the lowest prevalence of malnutrition, would have the lowest risk of developing konzo.

On the other hand, SAAs (especially methionine and cysteine) are important for the detoxification pathway transforming cyanide into the less toxic SCN excreted in urine [30]. However, the method used to assess cyanide exposure in vivo is the measurement of urinary SCN [29,31]. One could therefore question the accuracy of cyanide poisoning evaluation (with possible underestimation) in SAA-deficient individuals, such as konzo patients. Our study did not measure SAA, but confirmation of this assumption would challenge the abovementioned differential contribution of cyanide poisoning and malnutrition in the development of konzo, while calling for a more accurate procedure to evaluate cyanide poisoning in malnourished individuals. One way to do this would be to weigh or normalize measured urinary SCN by using a factor related to the lowered capacity of SCN synthesis in malnourished people.

When experimentally performed, the standard method reported for cassava processing in Idjwi proved to be efficient in lowering cyanide levels below the safety threshold after 4 days for both bitter and sweet cassava fresh roots. This observation is compatible with low cyanide content of flours measured in samples from the market in Bukavu, but contradictory with high urinary SCN levels found in participants from Idjwi. One explanation would be that the processing method was strictly followed for commercial cassava products, while shortcuts were introduced when processing cassava roots for domestic consumption. Unfortunately, we could not measure cyanide content of cassava species from household stocks. On the other hand, depending on the duration and storage conditions of cassava flours (which were not evaluated in this study), there could be an additional post-processing detoxification further lowering cyanide level in marketed cassava flour [32], even if there would have been shortcuts in root processing. This mechanism would not be prominently engaged in domestic stock, as they are consumed on a daily basis. Safe consumption of cassava products is important for preventing consequences such as endemic goiter and dysfunction in thyroid metabolism, but also outbreaks of konzo, if we consider that aggravation of the nutritional status in Idjwi would put the population at risk of developing konzo as stated above.

These results should be interpreted while taking into account the limitations of this study. First, the low number of konzo patients may have influenced the significance of some results. Replication in studies with a larger sample size would be appropriate. Second, given the study design (cross-sectional), this study could not evaluate the causal link between malnutrition and konzo, as it did not gather the information on the temporal relationship between malnutrition and konzo.

## 5. Conclusions

Our results show that cassava-derived cyanide toxicity does not necessarily coexist with konzo outbreaks. Nutritional status appears to be a key difference between populations undergoing konzo outbreaks and those without (respectively in Burhinyi and in Idjwi), despite similar levels of cyanide exposure. Therefore, in areas with a high prevalence of dietary cyanide poisoning, the nutritional status may be a determinant factor for konzo to appear. This observation opens a new avenue for research on konzo risk factors, to determine the specific role of malnutrition and further understand mechanisms underlying the disease. In particular, highlighting the prominent role of malnutrition and the potential existence of a transient triggering factor (possibly acute worsening of the nutritional status) puts malnutrition at the top of priorities when implementing preventive strategies for konzo. If we consider the population at risk for konzo (i.e., childbearing women, children and adolescents) and the high mortality due to direct and indirect consequences of malnutrition in the same population, any effort to fight against konzo would ultimately contribute to a more integrative public health policy against malnutrition. Thus, confirmation of our observations would not only constitute remarkable progress in the knowledge of konzo pathology, but it would significantly contribute to prevent konzo through identification of populations at risk based on new paradigms and in synergy with other health programs related to food safety.

## Figures and Tables

**Table 1:** Comparison of nutritional status between patients and controls from Burhinyi.

Parameters	Konzo Patients n (%)	Controls from Burhinyi n (%)	*p* ^1^
overall malnutrition (either wasting or stunting)			
Yes	9 (81.8)	9 (30.0)	0.0046
No	2 (18.2)	21 (70.0)	
acute malnutrition (wasting)			
Yes	6 (54.5)	1 (3.3)	0.0006
No	5 (45.5)	29 (96.7)	
chronic malnutrition (stunting)			
Yes	8 (72.7)	8 (26.7)	0.0119
No	3 (27.3)	22 (73.3)	

^1^ Fisher’s exact test.

**Table 2:** Comparison of nutritional status between controls from the 3 study areas.

Variables	Controls from Burhinyi n (%)	Controls from Idjwi n (%)	Controls from Bukavu n (%)	*p*
overall malnutrition (either wasting or stunting)				
Yes	9 (30.0)	12 (26.1)	10 (17.2)	0.341 ^1^
No	21 (70.0)	34 (73.9)	48 (82.8)	
acute malnutrition (wasting)				
Yes	1 (3.3)	3 (6.5)	4 (6.9)	0.902 ^2^
No	29 (96.7)	43 (93.5)	54 (93.1)	
chronic malnutrition (stunting)				
Yes	8 (26.7)	11 (23.9)	7 (12.1)	0.165 ^1^
No	22 (73.3)	35 (76.1)	51 (87.9)	

^1^ Pearson’s chi-squared test; ^2^ Fisher’s exact test.

**Table 3:** The differential contribution of the two risk factors of konzo in the three areas of South-Kivu.

Locations	Health Status	Prevalence of Risk Factors		
		Malnutrition		Cyanide Poisoning
		Wasting	Stunting	
Burhinyi	Konzo patients	High	High	High
	Controls	Low	Intermediate	High
Idjwi	Controls	Low	Intermediate	High
Bukavu	Controls	Low	Low	Low

## Data Availability

The data presented in this study are available upon request from the corresponding author.
